# Expanded Newborn Screening and Genomic Sequencing in Latin America and the Resulting Social Justice and Ethical Considerations

**DOI:** 10.3390/ijns7010006

**Published:** 2021-01-21

**Authors:** Juan F. Cabello, Fernando Novoa, Hanalise V. Huff, Marta Colombo

**Affiliations:** 1 Instituto de Nutrición y Tecnología de los Alimentos (INTA), University of Chile, Santiago 7830489, Chile; jfcabello@gmail.com; 2 Pediatric Neurology Fellowship Program, University of Valparaíso, Valparaiso 2341131, Chile; 3 Boston Children’s Hospital, Harvard Medical School, Boston, MA 02115, USA; hanalise.huff@childrens.harvard.edu

**Keywords:** ethics, Chile, Latin America, newborn genome sequencing, newborn sequencing

## Abstract

Newborn screening (NBS) has widely been utilized in developed countries as a cost-effective public health strategy that reduces morbidity and mortality. Developing countries, however, are new to the NBS scene and have their own unique challenges, both in instituting the program as well as effectively acting on the results. NBS offers numerous ethical issues on a global scale, however, here we argue that there are unique ethical issues surrounding the development and expansion of newborn screening in Latin America given its highly heterogenous population. Once a NBS program is effectively instated, ethical considerations continue when pursuing expansion of screening to include further conditions. While Latin America grapples with the ethics of expanded newborn screening (ENBS), some developed countries discuss utility of genomic sequencing technologies in the newborn population. When the ability to detect further pathology is expanded, one must know what to do with this information. As rare diseases are identified either on ENBS or via genome sequencing, access to treatments for these rare diseases can be a real challenge. If we consider newborn screening as a global initiative, then we need more than a deontology approach to analyze these challenges; we need an approach that considers the unique characteristics of each territory and tremendous heterogeneity that exists prior to the implementation of these programs. As genomic technology advances further in the developed world, while some developing countries still lack even basic newborn screening, there is a further widening of the gap in global health disparities. The question is posed as to who has responsibility for these newborns’ lives on an international level. Without an approach towards newborn screening that accounts for the diverse global population, we believe optimal outcomes for newborns and families across the world will not be achieved.

## 1. Introduction

Newborn screening (NBS) is a widely used public health strategy that has demonstrated high-cost effectiveness by reducing morbidity and mortality [1,2]. By focusing on diagnosis in the newborn period, there is a goal of initiating therapeutic intervention prior to irreversible damage, thereby dramatically improving outcomes and reducing the burden of devastating diseases. While the initiation of lifelong therapies including pharmaceuticals and special diets can be very costly on a society, it has been shown that it is in fact a greater financial burden on society for this individual to have immense neurologic deficits and the inability to be a productive member of society. 

While some developed countries have expansive NBS programs and some programs have proposed newborn genome sequencing as a possibility in the future [2], some developing countries grapple with the institution of the initial NBS program. While there is a relationship between level of economic development of a country and the efficiency of NBS implementation, the fact that this strategy permits more efficient public resource usage has led to increased interest of lower income countries to accelerate their implementation. Latin America joined the NBS discussion 20 to 30 years after pioneer countries, allowing more informed decisions when choosing the technology and the list of conditions to include. Here we review the process by which Chile developed its NBS program and comment on the programs and lack thereof utilized by other Latin American countries. 

The institution of newborn diagnostics poses various ethical dilemmas, and it is argued that the ethical challenges within Latin America are different than those of the NBS pioneer countries. When compared to the NBS pioneer countries, Latin American countries have important health system, political and cultural differences that may impact the utilization as well as costs and benefits of expanded newborn screening and genomic technologies. Latin American countries may differ in their approach to patient autonomy, access to pharmaceuticals, distribution of resources, and access to specialists and programs that ensure the treatment of those diagnosed in the screen. These important differences may in fact alter the cost–benefit ratio of the institution of NBS and genomic programs in certain contexts. 

The ethical struggles often do not end with disease identification. The diagnosis of rare diseases presents a unique challenge for countries that lack infrastructure to research them and financial capability to fund the often highly-expensive treatment for their citizens. We argue the importance of considering the Latin American perspective as the developed world moves towards further advancement in the form of expanded newborn screening (ENBS) and integration of genomic technologies into common practice. 

## 2. Institution of NBS Programs 

### 2.1. Chile

According to data on child mortality, life expectancy and per capita income, Chile is among the countries with the best macroeconomic and health indicators within Latin America [3,4]. Despite this, there was a delay of more than 30 years in development of a newborn screen program following countries, such as the United States. This delay stemmed largely from skepticism of Chilean authorities regarding the existence of these seemingly rare diseases. In the 1980s, a group of health professionals published the utility of early treatment in patients with phenylketonuria (PKU) and congenital hypothyroidism (CHT). This NBS program was finally started in Chile in collaboration with the pioneers of the United States NBS program, such as Dr. Robert Guthrie, who personally trained the Chilean laboratory team in the implementation of NBS techniques. 

In 1989, the National Institute for Nutrition and Food Technology (INTA), in conjunction with the Santiago Western Health Service, initiated a pilot NBS program of PKU and CHT which covered 20% of the country’s newborn population, which is 230,000 live newborns every year [5]. At that time, convincing the authorities to start a NBS program was not easy given Chile had just recently defeated primary malnutrition as the main cause of infant mortality. Data were published establishing the prevalence of PKU and CHT in Chile to be 1:14,640 and 1:2000, respectively. This data enabled a favorable cost–benefit ratio to be established, further validating the utility of the NBS implementation as a national program. In 1992, Chile’s Ministry of Health ruled on the start of the NBS program for PKU and CHT, which was implemented in a step-wise fashion until all 15 regions of the country were covered. In 1998 the program managed to cover the entire national territory. To this day, this program has achieved the detection of more than 3000 patients with CHT and 500 with PKU within Chile. Treatment coverage with special food is lifelong, and a team of highly specialized professionals is available to monitor them within a centralized program that provides regular follow-up and has proven excellent outcomes [6]. 

An ENBS pilot program is underway currently to increase the number of pathologies from 2 to 26 conditions, thereby extending the benefit to a significant number of newborns. All 26 conditions included in the pilot ENBS are treatable via therapeutics or special diet. The pilot program was introduced in a step-wise fashion and is currently in phase one of four. Phase one includes newborns of one large center to ensure follow-up. As it currently stands, these 26 conditions are identified later in life clinically once they have already caused irreversible neurologic sequelae. By identifying the conditions at the newborn stage and preventing the clinical effects of the disease, we are not only reducing disease burden and improving quality of lives of patients and families, but also reducing public health care expenditures. Public funding already exists for the treatment of these 26 diseases once identified clinically, however, by identifying the pathology via ENBS prior to symptoms, the public funding for lifelong therapeutics is accessed and the neurologically intact individual can contribute productively to society.

Chile has benefited immensely thus far from the NBS from a morbidity and mortality perspective as well as a cost-effectiveness perspective. The country is excitedly optimistic that the ENBS will provide further benefits for individuals, families and the health care system as a whole. As infrastructure for this addition to the newborn screening routine is solidified, there will likely be natural progression towards further advanced technologies such as genomic screening; however, there is no plan to integrate these advanced and comparatively expensive technologies at a universal, publicly-funded level at this time. 

### 2.2. Latin America

Latin America is composed of countries that differ dramatically economically, culturally and politically, and thus, the region cannot be viewed as one homogenous entity. Within Latin America to this day, there are countries that have child mortality figures above 60 per 1000 live newborns, and poverty levels of about 40% [3,4]. In 2007, it was reported that less than 50% of newborns in the region were screened for some pathology [7]. Uruguay, Costa Rica, Cuba and Chile are noted for their establishment of NBS programs and for entering into ENBS [8]. Countries such as Brazil, Mexico and Argentina face the challenges of extreme heterogeneity between populations within each country. In Brazil, for example, regions deep in the Amazon struggle with providing basic NBS through public funding for newborns whereas in the wealthy aspects of Rio, families can pay out of pocket for genomic sequencing. Given the heterogeneities of the populations of these regions, there are often significant inequities in access to NBS and ENBS based on class system related health disparities. Other smaller countries within Latin America have unstable economies and government systems and therefore have no reported national screening programs. 

## 3. Ethical Considerations of NBS and Incorporation of Genomic Technologies

### 3.1. Chile and Latin America

Implementation of a NBS program in a country with high levels of poverty, child mortality and primary malnutrition is completely different than in one with greater levels of economic and political stability and existing public health funding programs. Resource distribution becomes a limiting factor in the former. A country that is still struggling with childhood death due to malnutrition is less likely to be focused on newborn screening. Governments often opt for curative and non-preventive health initiatives, thereby neglecting preventive initiatives such as the NBS. The political and social stability of the country has a direct effect on the possibility of an NBS expansion as well as advancement of other central issues to health care development. A country requires the infrastructure to be able to process and distribute results once collected as well as a program set up to act on the results [9]. Diagnosis of PKU, for example, offers little benefit if there is no support and education system for families to perform diet changes essential to change the child’s outcome. While some will discuss whether to initiate NBS for CHT, PKU or hemoglobinopathies in the first place, others will be discussing whether to expand the screening to 20 or 30 conditions, and others yet may discuss whether to incorporate genomics. The evidence of success demonstrated by the Chilean NBS and now ENBS pilot program will likely serve as both a model and inspiration for other Latin American countries going forward. 

The emergence of the NBS within Latin America highlights the important role of the health team as patient and human rights advocates. A large part of the success of the Chilean program lies in the ability of the health care providers to demonstrate the burden of the disease through data and prove the cost effectiveness of the program’s institution. It is, therefore, of utmost importance to positively influence local governments to implement and refine these public programs for the sake of our patients and their families. The role of patient advocate challenges us to be the voice of those who are not yet born, as well as that of a society that seeks equity of basic social rights such as health care.

Regional initiatives such as those within Latin America are essential as they encourage collaboration and assistance among countries of differing levels of cultural, political and economic stability. As physicians, we all aim to provide the best care and treatment for all of our patients and this care should not differ based on national origin. We argue that these international efforts should be coordinated by scientific societies and sustained by international collaborations. The exchange of experiences allows us to learn from each other and work together to save as many newborns from preventable multisystemic sequelae as possible, as we are all, ultimately, one global humanity. 

### 3.2. The United States and Developed Countries

As technology advances quickly and there is a barrage of new diagnostic information available to providers, developed countries face ethical challenges regarding how to apply this new technology. Within the realm of newborn diagnosis, genomic sequencing has been proposed for use as an alternative, as a supplement and as a potential replacement of current standard NBS in some developed countries. In the United States, for example, a study in California compared whole exome sequencing (WES) to traditional NBS by tandem mass spectrometry (MS/MS) to determine whether WES could replace the traditional method. It was found that sensitivity and specificity of WES was in fact lower than the MS/MS method but there was possible utility of WES as a second tier [10]. In Norway, a study looked at the utility of Next Generation Sequencing (NGS) as a second tier to supplement NBS and showed benefit [11]. Pilot programs in the United States at several different sites used various genomic sequencing technologies to screen for conditions beyond the traditional NBS and looked at the utility both in populations of sick newborns and in those that are healthy [12]. 

Various ethical issues arise with these proposed new programs, such as the dilemma of how to communicate the genomic results to families and what to do with this information especially in the case of variants of unknown significance [13]. Further ethical discussion of these genomic technologies is beyond the scope of this paper. What we propose, however, as a relevant ethical dilemma is the advancement and implementation of these technologies in certain countries while basic screening in other countries has yet to be achieved. The use of genomic technologies expands beyond the goal of just decreasing infant morbidity and mortality and has the potential to further expand health disparities in different social and racial groups due to inequity in access to these techniques. We believe improving social and racial inequities through universal coverage programs emerges as a priority [14]. 

### 3.3. Global Ethical Considerations

Globally, the challenges are even greater. From an ethical approach, we might consider it fair to assume that if a human being is born in a developed country, then he or she will have mandatory screening of more than 40 different conditions, with some employing genomic technologies, and be given treatment that can both save his or her life and avoid disabling neurological sequelae. On the other hand, if this same human being is born in a country of less fortunate economic circumstances, the child may fall victim to consequences such as disability or even death. Despite scientific breakthroughs allowing diagnosis and treatment of now preventable metabolic illnesses, newborns still die globally based solely on the country they are born into. The dilemma of who the weight of responsibility of a preventable death should lie on is a great one. Should the wellbeing of this child be the responsibility of the parents, the state where this child is born or is it the responsibility of humanity as a whole? We argue that the global public health community should address as many newborns as it can to lobby for ENBS across the globe to reduce global morbidity and mortality due to preventable outcomes. 

The new genomic technologies pose an important financial ethical dilemma that may perpetuate global inequities in newborn outcomes globally. In countries like the United States, some health insurance companies cover the costs of novel diagnostic techniques. Given economic and political circumstances, many Latin American countries are unable to tap into this technology solely for this reason. It can be argued that inequity in access to health is not just a local challenge but a global problem. If "global economies" are already being raised as initiatives to reduce these inequities, we argue that prioritizing NBS implementation globally should be one of the first points addressed.

### 3.4. Ethical Considerations When Approaching Rare Diseases

The ethical dilemmas of newborn pathology do not end with diagnosis. Upon diagnosis, the next important dilemma is setting the patient and family up with the best therapeutic strategy, which in some cases, may be quite costly. The source of funding for these therapeutic strategies is another ethical challenge that disproportionately effects developing countries. When the disease diagnosed is considered a rare disease, there is even less chance of a financially feasible therapy.

Many years have passed since governments have become aware of the characteristics and situation of patients with rare diseases yet many still die or live in very poor conditions due to lack of diagnosis and lack of treatment access. From an ethical point of view, these patients pose a complex dilemma of justice in which it is necessary to harmonize the rights of each individual to medical assistance and access to validated treatments with the rights of other patients with different conditions that also require high-cost treatments in resource-limited settings. In addition, it is necessary to make a thorough analysis of the efficacy and cost–benefit ratio of the different therapeutic alternatives available if a condition is identified through newborn screening with or without sequencing. One aspect that illustrates the difficulties that exist in the field of justice in this area, is the fact that drugs to treat this type of disease should it be detected through NBS technologies have had less development and production because they are not economically profitable for researchers especially in resource-limited settings.

For this same reason, there is a lack of motivation in researching drugs for very rare diseases, which is why they are known as "orphan drugs." The concept of “orphan drugs” originated in the 1980s with culmination in the Orphan Drug Act devised in the United States in 1982 [15]. This situation requires states to offer incentives to stimulate this area of research. Currently, there is limited availability of drugs but a high need resulting in an inability to provide optimal care to all patients. The balance of financial commitment to development of drugs for rare diseases identified through newborn screens with the financial and moral burden of the suffering of these untreated children with rare diseases is an important ethical conundrum. It is necessary also to rationalize their use and establish criteria to decide whether all those carriers of rare diseases detected through genetic technology deserve treatment or whether only those which will achieve a relevant benefit in quality of life and survival should be treated. 

## 4. Discussion

In the lottery of nature, some persons are born equipped with special talents that facilitate their development while others are born with important deficiencies that will impede their ability to survive and thrive if they do not receive adequate treatment after correct diagnosis. Among these individuals are those with so-called rare diseases. These individuals, if not correctly diagnosed in the newborn period, will go on to have significant morbidity and mortality and result in a significant burden on families as well as society as a whole. With NBS, and particularly with genome-based technologies, there is the ability to now identify and treat many rare diseases prior to symptoms and therefore prior to lasting neurologic damage. It has been demonstrated through cost–benefit analysis that despite the large expenses of the lifelong therapies needed for these identified individuals, the overall cost is less than the financial burden alone of a neurologically devastated member of society, not to mention the overall benefit to the individual, their family and the community.

With this new technology, however, comes new ethical implications based in global inequities in access to both the diagnostic techniques as well as the therapeutic strategies. Here we highlight the wide spectrum of access to newborn diagnostic technologies depending on country of origin and socioeconomic status. Where countries in the developed world grapple with the new ethical dilemmas of introducing more thorough and expensive tests, such as genomic sequencing which can sometimes provide an excess of information with absence of treatment options, other countries struggle to impose basic NBS programs. 

From the point of view of currently accepted ethical principles, it is a matter of deciding whether NBS for every newborn regardless of country origin is a matter of fairness (principle of first order) or a matter of charity (principle of second order). It is necessary to bear in mind that the decisions for the benefit of each patient always includes the global care of the patient and family, with or without genome-based technologies.

Rare diseases are unique in that while they are less frequent than other diseases, they present a burden of disease much higher than that of other more frequent diseases and secondary to this, require such costly treatments that patients cannot assume the financial burden individually. In the past, access to health was mainly assumed on an individual basis. With the progress of society, the previously unrelated realms of politics and health were united, forming health policies that allowed more equal access for people to adequately treat their diseases. As a result, it can be argued therefore, that this financial burden should be a function and responsibility of the State [16].

It can be argued that the institution of NBS in many countries was a way to improve equity within a society by allowing every newborn to have access to screening and therefore early identification and treatment of potentially devastating diseases, thereby preventing the negative effects of the disease regardless of socioeconomic status. Unfortunately, as new diagnostic technologies arise, such as the expansion of the NBS as well as genome sequencing, inequities both within countries and between the developed and developing world can in fact be increased. Technology has increased to the point that some newborns, when born into wealthy families within wealthy societies, can have a barrage of tests done often with information with unknown significance, whereas a similar newborn when born into a less financially fortunate circumstance would be unable to even have basic screening for treatable diseases. Though medical advances in diagnostic technology is important and essential for overall global progress, it is important to always consider the ethical implications of these advances and the possibilities, as in the case of ENBS and genomics, the perpetuation of global inequities.

## 5. Conclusions

The NBS is a tool that for decades has been shown to be cost effective in preventing mortality and morbidity, thus achieving health goals across the world. By identifying treatable diseases before they have produced their devastating and permanent effects, one not only reduces the overall cost on society of supporting a neurologically devastated child possibly into adulthood, but also provides an immense improvement in quality of life for the individual, their family and the community. In an ideal world, all countries, regardless of economic status, would have access to this vital program. This, however, is not the case, resulting in global inequities in access to lifesaving diagnostics and therapeutics. A primary goal of NBS and ENBS is to identify only diseases for which there is treatment and where a measurable impact can be made in cost to quality of life by early identification and treatment. Genomic sequencing, while sometimes used in a targeted manner or as a confirmatory test or second tier, can other times identify diseases and unknown mutations for which a treatment is lacking. Exploring the utility of newborn genomic screening technologies in certain countries while other countries lack the capability of identifying treatable diseases such as PKU and CHT perpetuates global inequities in health outcomes.

We encourage the global medical community to look at the ethical issues associated with NBS, ENBS and genomics from a perspective outside the developed world. We suggest working towards a world where all countries regardless of economic status can provide basic NBS with access to therapeutics upon diagnosis prior to engaging in further diagnostic advancements which perpetuates heath inequities globally. As we are all one global humanity, we believe we must all work together to increase access to care and improve health outcomes for all newborns regardless of origin. 
